# Polyphasic characterisation of *Microcoleusautumnalis* (Gomont, 1892) Strunecky, Komárek & J.R.Johansen, 2013 (Oscillatoriales, Cyanobacteria) using a metabolomic approach as a complementary tool

**DOI:** 10.3897/BDJ.11.e100525

**Published:** 2023-04-21

**Authors:** Ivanka Teneva, Detelina Belkinova, Tsvetelina Paunova-Krasteva, Krum Bardarov, Dzhemal Moten, Rumen Mladenov, Balik Dzhambazov

**Affiliations:** 1 Faculty of Biology, Plovdiv University “Paisii Hilendarski”, Plovdiv, Bulgaria Faculty of Biology, Plovdiv University “Paisii Hilendarski” Plovdiv Bulgaria; 2 Institute of Biodiversity and Ecosystem Research, Bulgarian Academy of Sciences, Sofia, Bulgaria Institute of Biodiversity and Ecosystem Research, Bulgarian Academy of Sciences Sofia Bulgaria; 3 The Stephan Angeloff Institute of Мicrobiology, Bulgarian Academy of Sciences, Sofia, Bulgaria The Stephan Angeloff Institute of Мicrobiology, Bulgarian Academy of Sciences Sofia Bulgaria; 4 InoBioTech Ltd., Sofia, Bulgaria InoBioTech Ltd. Sofia Bulgaria

**Keywords:** Cyanobacteria, *Phormidiumautumnale*, polyphasic, morphology, ultrastructure, TEM, 16S, phylogeny, metabolomics, biochemical markers

## Abstract

As a result of the continuous revision of cyanobacterial taxonomy, *Phormidiumautumnale* (Agardh) Trevisan ex Gomont, 1892 has been transferred to the genus *Microcoleus as Microcoleusautumnalis* (Gomont, 1892) Strunecky, Komárek & J.R.Johansen, 2013. This transfer was based on a single strain and literature data. In the present study, we revise the taxonomic position of *Microcoleusautumnalis* by applying the classical approach of polyphasic taxonomy and additionally using metabolomics. Cyanobacterial strains identified as *Phormidiumautumnale* and *Microcoleusvaginatus* (type species of the genus *Microcoleus*) were used for comparative analyses. In addition, the taxonomic relationship between the species *Phormidiumautumnale* and *Phormidiumuncinatum* was determined on the basis of polyphasic characteristics. Monitoring of the morphological variability of *Phormidiumautumnale* and *Microcoleusvaginatus* strains showed a difference in the morphology concerning the ends of the trichomes, the shape of the apical cells, as well as the presence/absence of the calyptra and its shape. The performed TEM analysis of the thylakoid arrangement of the studied strains showed parietal arrangement of the thylakoids in the representatives of genus *Phormidium* and fascicular arrangement in genus *Microcoleus*. Molecular genetic analyses, based on 16S rDNA, revealed grouping of the investigated *P.autumnale* strains in a separate clade. This clade is far from the subtree, which is very clearly formed by the representatives of the type species of genus *Microcoleus*, namely *M.vaginatus*. The metabolomic analysis involving *P.autumnale* and *M.vaginatus* strains identified 39 compounds that could be used as potential biochemical markers to distinguish the two cyanobacterial species. Based on the data obtained, we suggest changing of the current status of *Microcoleusautumnalis* by restoring its previous appurtenance to the genus *Phormidium* under the name *Phormidiumautumnale* (Agardh) Trevisan ex Gomont, 1892 and distinguishing this species from genus *Microcoleus*.

## Introduction

In recent years, the taxonomy and systematics of the phylum Cyanobacteria have been actively revised and reorganised, based on new data gained mainly from different molecular genetic studies (Komárek 2006, Komárek et al. 2014, Komárek 2016, Strunecký et al. 2022). Such a process is typical for cyanobacteria, but it also affects a number of other plant, fungal and animal taxa. The problem is that sometimes as the established rules (when they exist) are interpreted in a subjective way and often giving priority to the new features, we neglect the well-functioning old, which in most cases are traditionally established and accepted (Komárek 2020).

In the taxonomy of cyanobacteria, the polyphasic approach is most often applied (Anagnostidis and Komárek 1985, Hoffmann et al. 2005). This approach combines molecular-genetic, morphological, ultrastructural, biochemical and environmental data, with priority given to the molecular genetic data, while others are considered complementary (Komárek 2009, Komárek 2016). What happens in practice? Based on mainly molecular genetic data combined in most cases only with cytomorphological features, new genera are separated (often with only 2-3 representatives) and widespread species are renamed. There is no complete set of data to confirm and convincingly show the need for this change (Komárek 2020). Even assuming that the principles of polyphasic taxonomy are followed, it should be kept in mind that the 16S rDNA sequence is not a marker that allows for subgeneric identification and that the use of other genetic markers in solving taxonomic cases should not be ignored (Moten et al. 2017).

The presence of crypto- and morphospecies amongst representatives of the Cyanobacteria should not be overlooked. The use of complete morphological, ultrastructural, biochemical and ecological data should be a requirement in determining the taxonomic position of a certain taxon. Otherwise, taxonomic changes may occur that are contradictory and not sufficiently justified.

The main targets are polyphyletic genera, such as *Phormidium*, *Microcoleus* and *Leptolyngbya* and taxa in which morphological criteria overlap and are not sufficiently descriptive to make definite decisions. The current study was provoked by another taxonomic change related to the species *Phormidiumautumnale* (Agardh) Trevisan ex Gomont, 1892, which was renamed in 2013 to *Microcoleusautumnalis* (Gomont, 1892) Strunecky, Komárek & J.R.Johansen, 2013 (Strunecký et al. 2013).

Both genera, *Phormidium* Kütting ex Gomont and *Microcoleus* Desmaziéres ex Gomont, are polyphyletic and rich in species within the order Oscillatoriales (Palinska et al. 2011, Stoyanov et al. 2014). They are amongst the earliest described genera of order Oscillatoriales (Gomont, 1892). The type species of the genus *Phormidium* is *P.lucidum* Kützing ex Gomont (Geitler 1942) and the type species of the genus *Microcoleus* is *M.vaginatus* (Vaucher) Gomont (Geitler 1942, Drouet 1968). Although there is an available 16S rDNA sequence from the type species *P.lucidum* in the GenBank, in the last updated classification of cyanobacterial orders and families, it is noted that reliable sequencing data for the type species of genus *Phormidium* (*P.lucidum*) are missing and/or their phylogenetic placement is ambiguous (Strunecký et al. 2022). *Phormidium* is one of the most difficult cyanobacterial genera from a taxonomic point of view (Komárek and Anagnostidis 2005). It consists of numerous morphotypes with many transitional forms. The relatively wide range of different morphological species previously assigned to the genera *Phormidium*, *Oscillatoria* and *Lyngbya* were reorganised by Komárek and Anagnostidis (2005) into eight different morphological groups, which differ in the morphology of the apical ends of trichomes.

The difficulty in distinguishing between the genera *Phormidium* and *Microcoleus* using the classical approach comes from the lack of sufficiently descriptive morphological criteria. According to the literature, *P.autumnale* and *M.vaginatus* do not differ in cell size. The range of variation in the length and width of their cells overlap. According to Strunecký et al. (2013), the morphological difference between *P.autumnale* and *M.vaginatus* is only in the form of colonies and organisation of the filaments, but the morphology of the trichomes is very similar (Strunecký et al. 2013). There is a difference in their habitats. *P.autumnale* is a freshwater species distributed mainly in streams, rivers and waterfalls, but also amongst the overgrowth (periphyton) on underwater substrates. The main habitat of *M.vaginatus* is the soil.

The situation is more complicated because the data obtained by molecular approaches are often inconsistent with the taxonomy based on the morphological studies (McAllister et al. 2016). Marquardt and Palinska showed that cyanobacterial strains morphologically assigned as *P.autumnale* are genetically different and grouped into different clusters (Marquardt and Palinska 2007). Studies of cyanobacterial blooms in New Zealand, based on the polyphasic approach, have reported dominance of genus *Phormidium* and presence of significant morphological variability between the blooming strains (*P.autumnale* and *P.uncinatum* Gomont ex Gomont, 1892), while other studies of the same strains, based on 16S rDNA, have identified *P.autumnale* as the dominant species (Heath et al. 2010, Harland et al. 2014, McAllister et al. 2016). These results unequivocally show that further in-depth studies of the genus *Phormidium* and, in particular, *P.autuamnale* are required.

However, it is clear that this cannot happen, based only on morphology and molecular genetic criteria. The inclusion of ultrastructural characteristics (e.g. thylakoid arrangement) as well as biochemical criteria (specific metabolites) in the process of characterisation of these taxa would help to clarify this problem. Thylakoid models are recognisable and useful in distinguishing morphologically simple single-celled and filamentous species. In addition, the various modifications in the arrangement of thylakoids are apparently related to the cryptogenera, in which their ultrastructural modification may correlate with the phylogenetic position (Komárek 2016, Mareš et al. 2019).

The biochemical criteria in general have always been poorly represented in the polyphasic characterisation of cyanobacteria. The reason is, on the one hand, insufficient scientific information and, on the other, the unclear taxonomic value of these criteria. Thus, in our opinion, the demonstration of the applicability of metabolic analyses for polyphasic characterisation of cyanobacteria is very useful. Studying the metabolites of *P.autumnale* and *M.vaginatus* strains, here we demonstrate the possibility of using metabolic analyses for polyphasic characterisation of cyanobacteria.

In the present study, applying the classical polyphasic approach and metabolomic analysis, we showed that *P.autumnale* and *M.vaginatus* belong to different genera and the classification of *Phormidiumautumnale* as *Microcoleusautumnalis* is incorrect. In addition, based on the polyphasic characterisation, we determined that the studied strains of *P.autumnale* and *P.uncinatum* are different species belonging to genus *Phormidium*.

## Materials and methods

### Strains and culture conditions

A total of 11 cyanobacterial strains from three collections were used in the present study: seven strains from the Plovdiv Algal Culture Collection (PACC), Paisii Hilendarski University of Plovdiv, Bulgaria; three strains from the Culture collection of Autotrophic Organisms (CCALA) of the Institute of Botany of the Czech Academy of Sciences, Třeboň and one strain from the Culture Collection of Algae (SAG) at the University of Göttingen, Germany. Cyanobacteria were cultured for 1 month under sterile conditions (75 cm^2^ culture flasks, TPP, Trasadingen, Switzerland) in liquid alkaline Z-nutrient medium (Staub 1961), with a photoperiod of 12 h/12 h light/dark, at a light intensity of 10 µmol photons s^-1^ m^-2^ provided by 40 W cool-white fluorescent lamps. This cultivation was carried out in order to accumulate the cyanobacterial mass necessary for the study (morphological analysis, transmission electron microscopy (TEM), preparation of extracts, DNA isolation and molecular genetic analyses.

### Morphological analysis

Investigated cyanobacterial strains belong to three species: *Microcoleusautumnalis* (Gomont) Strunecky, Komárek & J.R.Johansen 2013 (previously *Phormidiumautumnale* (Agardh) Trevisan ex Gomont 1892), *Microcoleusvaginatus* Gomont ex Gomont 1892 and *Phormidiumuncinatum* Gomont ex Gomont 1892. Data for the strains originally identified as *Phormidiumautumnale* and *Phormidiumuncinatum*, as well as for the *Microcoleusvaginatus* strains, are presented in Table 1.

Morphological analyses were performed using a Magnum-T microscope equipped with a high-resolution 3 Mpx Si-3000 XLiCap digital camera and software (Medline Scientific Ltd., Chalgrove, UK). In the course of the work, photo documentation of the examined samples was also performed. At magnifications of 100÷1000×, the variability of the following phenotypic features during the exponential growth phase of the strains was monitored: shape of the filaments; sheaths - presence and condition; trichomes - colour, shape of the trichome ends, mobility, presence/absence of granulations; presence of constrictions at the cross-walls, shape of the cells; apical cell of the trichome - shape, calyptra (presence/absence, shape of the calyptra). Cell measurements: length (L) and width (W). The measurements were performed on a minimum of 50 cells.

### Transmission electron microscopic (TEM) analysis of thylakoid arrangement in the cells of selected strains

Cultured strains were harvested by centrifugation at 3000×g for 5 min. Cyanobacterial filaments were washed with 0.1 M cacodylate buffer and fixed with 4% glutaraldehyde in 0.1 M cacodylate buffer at pH 7.2 for 4 h at 4°C. Then, the samples were washed three times with 0.1 M cacodylate buffer, after which they were fixed with 1% osmium tetroxide in 0.1 M cacodylate buffer at room temperature for 1 h. Cyanobacteria were pelleted by centrifugation and embedded in 1% agarose and cut into small cubes. Dehydration was done in an ascending alcohol series: 30%, 50%, 70%, 90%, 95% ethanol for 15 min each, 100% ethanol (2×) for 30 min and propylene oxide once for 30 min and one more time for 15 min.

The dehydration was followed by impregnation with propylene oxide and resin (durcupan): propylene oxide:resin 2:1 for 30 min, propylene oxide:resin 1:1 for 30 min, propylene oxide:resin 1:2 for 30 min and pure resin overnight. The samples were polymerised at 56°C for 48 hours. Ultra-thin sections of 60–70 nm in size were cut using an ultramicrotome Reichert (Reichert-Jung Ultracut E Ultramicrotome, Optische Werke AG, Vienna, Austria). Sections were mounted on copper grids for electron microscopy and counterstained with 1% uranyl acetate in 70% methanol for 15 min, followed by Reynold's lead citrate for 20 min (Reynolds 1963). The prepared sections were examined in a high-resolution transmission electron microscope HR STEM JEOL JEM 2100 (JEOL Ltd., Tokyo, Japan) operating at 200 kV, equipped with a CCD camera GATAN Orius 832 SC1000 (Gatan GmbH, München, Germany).

### DNA isolation, PCR amplification and sequencing

Genomic DNA was extracted from 40 mg of fresh cyanobacterial mass using the xanthogenate-SDS (XS) extraction protocol of Tillet & Neilan (Tillett and Neilan 2001) or by the Proteinase-K extraction assay. DNA concentration and its purity were measured using a NanoDrop 2000 UV-VIS spectrophotometer (Thermo Fisher Scientific, Wilmington, DE, USA). The isolated DNA was visualised on an agarose gel by ethidium bromide and UV transillumination (MiniBIS Pro gel documentation system, DNR Bio-Imaging Systems Ltd., Jerusalem, Israel). 16S rDNA was amplified by using the primers 16S-16C-R (5’-AAGGAGGTGATCCAGCCGCA-3’) and 16S-1R-F (5’-AGAGTTTGATCCTGGCTCAG -3’) (Wilmotte et al. 1992, Seo and Yokota 2003 A PuReTaq™ ReadyToGo Beads kit (GE Healthcare, Buckinghamshire, UK) was used for the PCR reaction, including 1.5 U Taq DNA polymerase, 10 mM Tris-HCl pH 9, 50 mM KCl, 1.5 mM MgCl_2_ and 200 µM dNTP. Five pmol of both primers, 100 ng genomic DNA and DEPC-water were added to the mix for each reaction to a final volume of 25 µl.

Amplification was carried out in a TC-412 thermocycler (Techne, Cambridge Ltd., UK) using the following programme: DNA denaturation for 5 min at 94°C, followed by 30 cycles of 60 s at 95°C, 60 s at 53°C (hybridisation) and 2 min at 72°C (elongation). The reaction was completed with an elongation step of 10 min at 72°C. The obtained PCR-products were analysed by electrophoresis in a 1.5% agarose gel in Tris-Acetate-EDTA buffer (TAE). GeneRuler™ 100 bp DNA Ladder Plus (Thermo Fisher Scientific Baltics UAB, Vilnius, Lithuania) was used as a size marker. Gels were visualised with ethidium bromide and UV light.

After visualisation, the correct PCR products were excised from the gel and the isolated DNA was purified using a PureLink™ PCR Purification Kit (Thermo Fisher Scientific Baltics UAB, Vilnius, Lithuania). Purified 16S rDNA products were sent for sequencing to Eurofins Genomics Germany GmbH (Ebersberg, Germany). Sequencing was conducted by using the same primers as for the PCR amplification. Obtained 16S nucleotide sequences were compared with the available 16S sequences for other cyanobacterial strains in the NCBI database using BLAST (https://blast.ncbi.nlm.nih.gov/, accessed on 11 November 2022). All new 16S rDNA sequences from this study were deposited in the GenBank (National Center for Biotechnology Information, NCBI) under accession numbers OP626168 – OP626173 (OP626168 for *Lyngbyaaerugineo-coerulea* PACC 8601, currently *Potamolyneaaerugineo-caerulea*; OP626169 for *P.autumnale* PACC 5505; OP626170 for *P.autumnale* PACC 5511; OP626171 for *P.autumnale* PACC 5517; OP626172 for *P.autumnale* PACC 5527; OP626173 for *P.autumnale* PACC 5529).

### Phylogenetic analyses

For the purposes of the phylogenetic analyses, 16S rDNA sequences of identified and determined at the species level representatives of the genera *Phormidium*, *Microcoleus*, *Oscillatoria*, *Arthrospira*, *Kamptonema*, *Trichodesmium*, *Dapis*, *Thychonema*, *Wilmottia*, *Capilliphycus*, *Neolyngbya* and *Affixifilum* were retrieved from the NCBI database. Thus, the sequences of those members determined only at the generic level were not included in the analyses.

The multiple alignment of the selected nucleotide sequences (106 sequences with 1531 nucleotide sites) was carried out by the MAFFT version 7 (Katoh et al. 2017) (https://mafft.cbrc.jp/alignment/server/, accessed on 5 December 2022). Phylogenetic analyses were performed by using Maximum Likelihood (ML) and Neighbour-joining (NJ) methods with MEGA 7 (Kumar et al. 2016) and Bayesian approach with MrBayes v. 3.2.7a (Ronquist et al. 2012). The search for the best fitting models, which is a part of the phylogenetic software package MEGA 7 (Kumar et al. 2016), indicated that the Kimura 2-parameter model (K2+G+I) (Kimura 1980) is the most suitable for the analyses. This model was applied in the Maximum Likelihood (ML) and Neighbour-joining (NJ) analyses with four rate categories of the gamma distribution. The Bayesian estimation of phylogeny (Huelsenbeck and Ronquist 2001, Ronquist et al. 2012) was performed with MrBayes v. 3.2.7a on XSEDE (CIPRES, https://www.phylo.org, accessed on 5 December 2022). Two runs of eight Markov chains were calculated for ten million generations with sampling every 1000 generations. The first 25% of the sampled trees were discarded as burn-in. Consensus phylogenetic trees were reconstructed using the MEGA 7 software. All analyses were performed with 1000 bootstrap repetitions with a total of 1531 positions in the dataset. *Gloeobacterviolaceus* (FR798924) was used as an outgroup to root the trees.

### Metabolomic analysis

Biomasses (500 mg) from three *P.autumnale* strains (PACC 5522, PACC 5527, PACC 5529) and three *M.vaginatus* strains (CCALA 145, CCALA 152, CCALA 757) were used for extraction of polar and non-polar metabolites. The extraction procedures and LC-MS analysis were carried out as previously described (Teneva et al. 2022). Briefly, freeze-dried cyanobacterial biomasses from the selected strains were mixed with 3 ml MeOH followed by an ultrasonic bath extraction (Branson 5510R-DTH, Wilmington, NC, USA) for 20 min and consequently, 6 ml of chloroform (for 20 min on a shaker) and 3 ml of Milli-Q water were added. After centrifugation at 4000 rpm for 20 min, the methanol/chloroform fractions were collected and filtered through 0.20 µm Millex-FG hydrophobic PTFE filters (Merck KGaA, Darmstadt, Germany). Only methanol/chloroform fractions (containing non-polar compounds) were used for the LC-MS analysis.

Two microlitres of each of the fractions were analysed on a Q Exactive LC-MS/MS system (Thermo Fisher Scientific, Waltham, MA, USA) composed from an Accela quaternary HPLC pump with an Accela autosampler and an HRMS Q-Exactive detector with H-ESI electrospray. The reverse phase (RP) chromatographic separation was performed on a Kinetex EVO C18 150 mm × 3 mm, 2.6 µm core-shell column (Phenomenex Inc., Torrance, CA, USA). Mobile phases, mass spectral conditions and data treatment are described in detail by Teneva et al. (2022).

MS/MS spectra for annotated compounds with significant fold changes (analysed by the Perseus framework of the MaxQuant proteomics software package, https://maxquant.net/maxquant/, accessed on 11 November 2022) and acceptable p-value (< 0.05) between selected strain groups (*P.autumnale* and *M.vaginatus*) were subjected to a FISh coverage processing, SIRIUS MS/MS processing (https://bio.informatik.uni-jena.de/software/sirius/, accessed on 11 November 2022) and MS Finder Search (http://prime.psc.riken.jp/compms/msfinder/main.html, accessed on 11 November 2022). A limited number of compounds were validated manually by comparison with experimentally obtained or simulated MS/MS spectra from the METLIN script (Guijas et al. 2018) and MZ cloud databases, if available. Any data processing of metabolites outside Compound Discoverer was made using Xcalibur™ 2.2 (Thermo Fisher Scientific, Hemmel, UK).

### Statistical analysis

Data (excluding metabolomics) were presented as mean ± standard deviation (SD). Differences between the samples were evaluated by analysis of variance (ANOVA) and considered significant when p < 0.05. Quantitative MS data were statistically analysed and visualised by using the Perseus software package (https://maxquant.net/perseus/, accessed on 11 November 2022). Hierarchical clustering analysis and heat map were applied to group the quantified compounds, based on their abundance after Z-score normalisation and subtraction of mean values. Two-sample t-tests, combined with permutation False Discovery Rate (FDR) to correct for multiple testing, were used. Volcano plot display was used to visualise data.

## Results

### Morphological analysis

By applying the principles of the morphological approach, a description of the studied strains and measurements of their cells were performed at the beginning of the study.

#### Morphological description of *Phormidiumautumnale* strains

Data from the performed morphological analysis are presented in Table 2.

**Figure 1.**
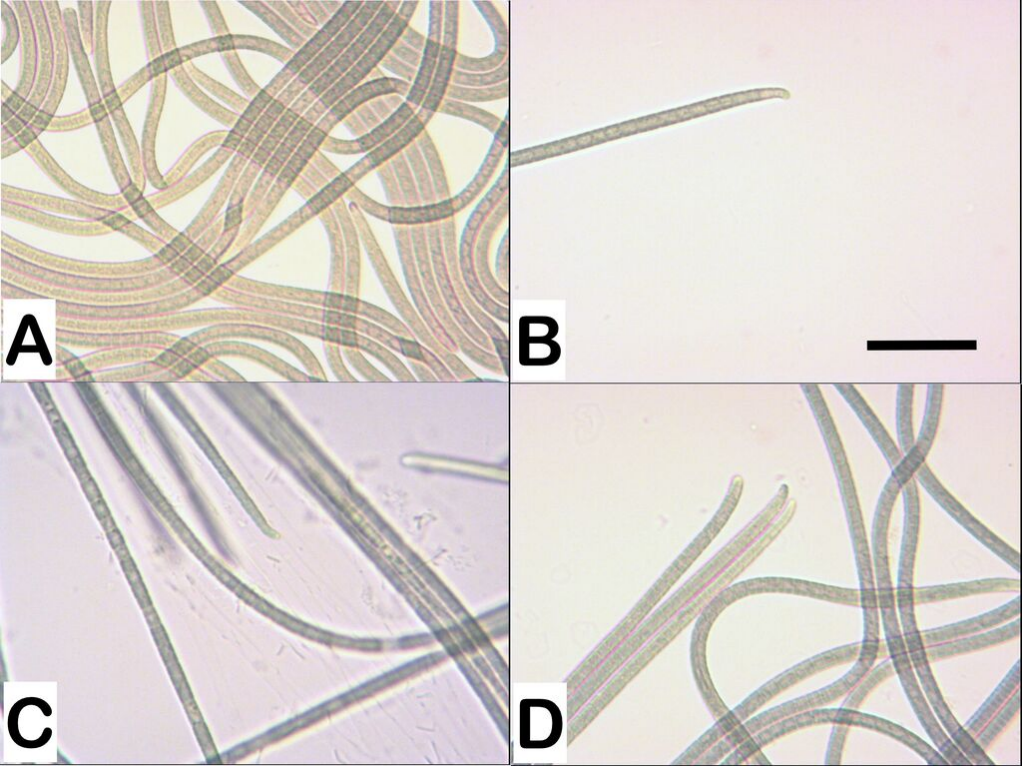
Photomicrographs of *Phormidiumautumnale* strains. **A, B** *P.autumnale* PACC 5517; **C** *P.autumnale* PACC 5527; **D** *P.autumnale* PACC 5529. Magnification 400×; Scale bar - 20 µm.

Thallus blue-green to dark greyish-green, forming a thin velvety membrane. Free-floating or attached to the walls of the culture flask, but also developing above the boundary of the membrane separating the nutrient medium from the air (aerophilic), forming creeping tufts. With ageing, the thallus detaches from the walls and floats in a common dark-green to yellowish-green mucilaginous mass on the surface of the culture flask. Filaments long, cylindrical ± straight or curved and tightly interwoven heterogeneous or ± parallel in places (Fig. 1A). Sheaths thin, mucilaginous, soft or clear, facultative, sometimes obscure or diffluent, colourless to amorphous, enclosing only one trichome.

Trichomes bright blue-green to yellowish-green, 3.3-4.0 µm wide (mean value), motile, slightly constricted at the granulated cross-walls, gradually attenuated towards ends (Fig. 1B-D). Cells usually shorter than wide, cylindrical to ± isodiametric (L/W = 0.6-1.0), with visible chromatoplasma and centroplasma or keritomised (Fig. 1D). Presence of necroidic cells. Apical cells elongated, rounded conical, curved, with rounded calyptra (Fig. 1B-D).

Specific characteristics: 1) Trichomes 3.3-4.0 µm wide (mean value), slightly constricted at cross-walls; cells short-cylindrical to ± isodiametric (L/W = 0.6-1.0). 2) Visible chromatoplasma and centroplasma or keritomised. 3) Trichome ends gradually and slightly narrowed. 4) Apical cells elongated, with a rounded conical shape, slightly curved. 5) Calyptra weakly expressed, with a rounded shape or absent.

#### Morphological description of *Microcoleusvaginatus* strains

Summarised data from the performed morphological analysis are presented in Table 3. Thallus bright olive-green, dark green to black, forming fascicles at the surface and walls of the culture flask. Old cultures form free-floating yellowish-green mucilaginous aerophytic or subaerophytic masses on the surface and separate yellowish aerophytic fascicles on the walls of the culture flask.

**Figure 2.**
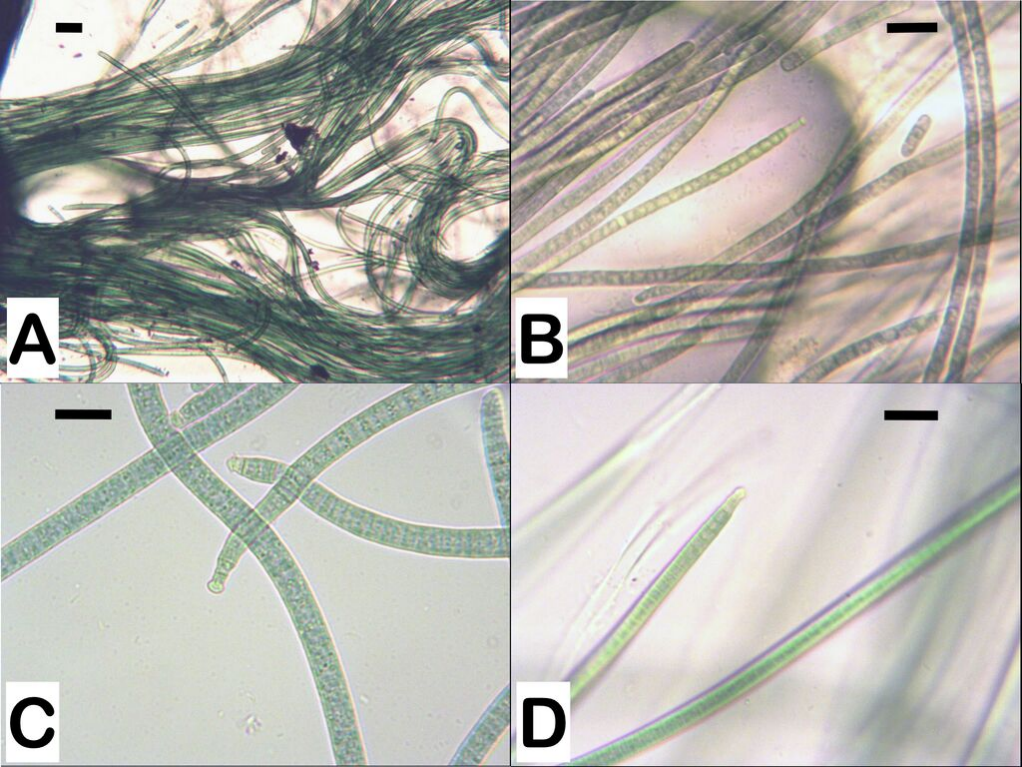
Photomicrographs of *Microcoleusvaginatus* strains. **A** *M.vaginatus* CCALA 757; **B** *M.vaginatus* CCALA 145; **C** *M.vaginatus* CCALA 152; **D** *M.vaginatus* SAG 2211. Magnification 400×; Scale bar - 10 µm.

Filaments long, cylindrical, straight or slightly curved, indiscriminately or in places ± parallel arranged (Fig. 2A). Sheaths thin, mucilaginous, clear, colourless, enveloping one trichome. Sometimes diffluent, forming a shapeless yellowish mass. Trichomes bright blue-green, with keritomised contents, 4.6-5.6 µm wide (mean value), motile, not constricted at the granulated cross-walls (Fig. 2C). The ends of the trichomes abruptly and strongly narrowed, curved to S-shaped contorted (Fig. 2C). The attenuation affects the last few cells, not just the apical one. Cells usually short cylindrical (L/W = 0.5-0.6), rarely ± isodiametric, 2.6-3.3 µm long. Presence of necroidic cells. Apical cells capitate, with conical, obtuse to hemispherical calyptra (Fig. 2C, D).

Specific characteristics: 1) Sheaths thin, mucilaginous, clear, colourless, enveloping one trichome. 2) Trichomes not constricted at cross-walls, 4.6-5.6 µm wide (mean value). 3) Trichome ends abruptly and strongly narrowed (last few cells), curved to S-shaped contorted. 4) Cells short cylindrical (L/W = 0.5-0.6), rarely ± isodiametric, keritomised. 5) Apical cells capitate, with conical, obtuse to hemispherical calyptra.

#### Morphological description of *Phormidiumuncinatum* PACC 8693

**Figure 3.**
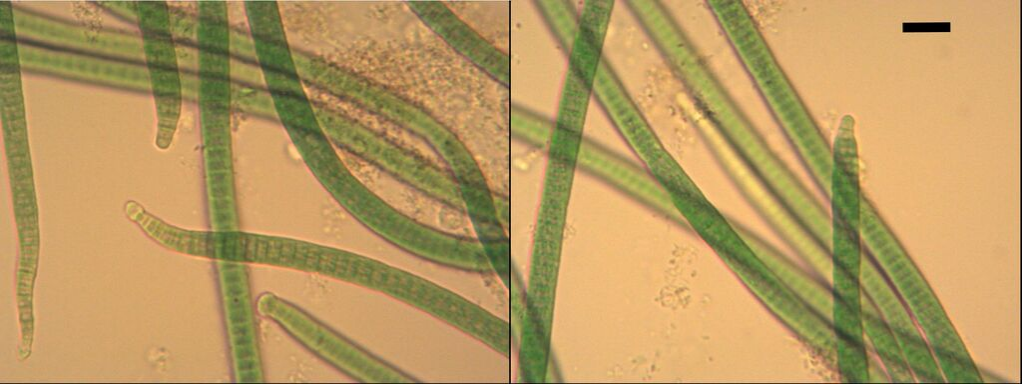
Photomicrographs of *Phormidiumuncinatum* PACC 8693. Magnification 400×; Scale bar - 10 µm.

Thallus bright blue-green, forming fascicles and tufts on the surface of the nutrient medium. Old cultures black-green, tufts retain their positions in the culture flask. Filaments long, cylindrical ± straight, indiscriminately or in places ± parallel arranged (Fig. 3). Sheaths thin, mucilaginous, soft, obscure or diffluent, colourless to amorphous. Trichomes bright blue-green, 6.0-9.0 µm wide (mean value), motile, not constricted or slightly constricted at the granulated cross-walls, abruptly narrowed towards the ends which are curved (Table 4). Cells short cylindrical, always distinctly shorter than wide (length ⅓ to ½ of the width), 1-4 µm long. Apical cells capitate, with rounded conical calyptra (Fig. 3).

Specific characteristics: 1) Trichomes 6-9 µm wide, not constricted or slightly constricted at the cross-walls, abruptly narrowed towards the ends; 2) Cells always short cylindrical (length ⅓ of the width); 3) Apical cells capitate, with rounded conical calyptra.

The culture strain corresponds phenotypically to *P.uncinatum*.

#### Cell sizes

According to literature data, the species *Phormidiumautumnale* and *Microcoleusvaginatus* do not differ in cell size. The range of variation in the length and width of their cells overlaps (2-4 × 4-7 µm and 2-5 × 3-7 µm, respectively). All the strains we examined, originally designated as *P.autumnale* and *M.vaginatus*, had similar cell sizes and fell within the range of variation of the two species. Results of the cellular measurements of the investigated strains are summarised in Table 5.

### Transmission electron microscopy (TEM) analysis

For decades, the thylakoid arrangement has been used in the classification of cyanobacteria as one of the key features for defining taxa. TEM analyses are becoming a regular part of the polyphasic characterisation of cyanobacteria, accounting for the fine structure of multiple strains. A recent comprehensive study by Mareš et al. (2019) mapped the ultrastructural data of more than 200 cyanobacterial strains and classified the thylakoid arrangement. Based on visual evaluation of the TEM dataset, the types of thylakoid arrangements were divided into eight categories: 1 - thylakoids absent, 2 - parietal, 3 - radial, 4 - fascicular, 5 - parallel, 6 - irregular, 7 - *Cyanothece*-like, 8 - unknown or ambiguous (Mareš et al. 2019).

**Figure 4.**
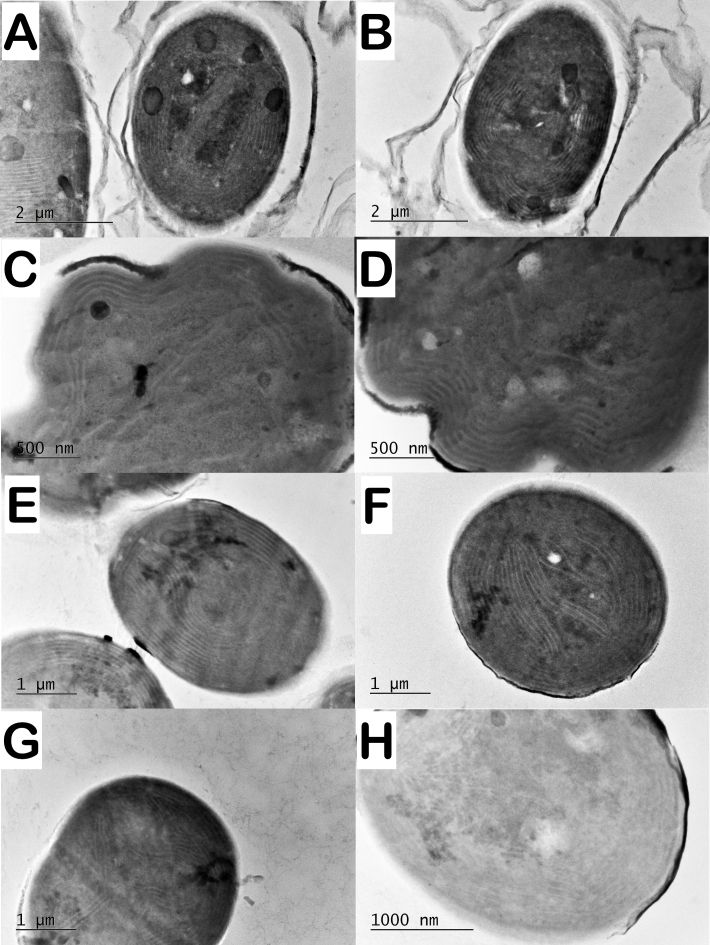
Ultrastructure of strains originally identified as *Phormidiumautumnale* with characteristic thylakoid arrangement. **A** parietal thylakoids with a central fascicle in *P.autumnale* PACC 5505; **B** parietal thylakoids composed of peripheral fascicles in *P.autumnale* PACC 5511; **C, D** parietal thylakoids in *P.autumnale* PACC 5522 (varies to simple parietal); **E, F** parietal thylakoids with a central fascicle in *P.autumnale* PACC 5527; **G** parietal thylakoids with a central fascicle in *P.autumnale* PACC 5529; **H** parietal thylakoids in *P.autumnale* PACC 5517.

In the strains of *Phormidiumautumnale* that we studied, the thylakoid system was organised more or less parietal (Fig. 4, Table 2). Thylakoids were usually aggregated parallel along the cell walls (Fig. 4), but often form central fascicles (Fig. 4F, G).

**Figure 5.**
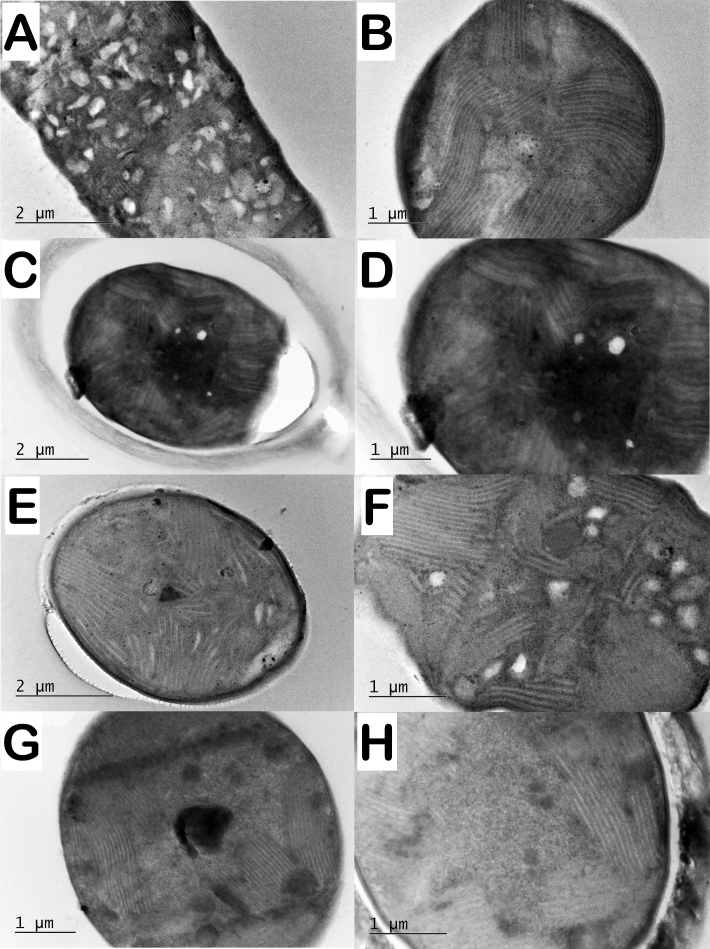
Ultrastructure of strains originally identified as *Microcoleusvaginatus* with fascicular arrangement of the thylakoids. **A, B** *M.vaginatus* CCALA 145; **C, D** *M.vaginatus* CCALA 152; **E, F** *M.vaginatus* CCALA 757; **G, H** *M.vaginatus* SAG 2211. **A** longitudinal section. **B-H** cross sections.

In contrast to the parietal arrangement of thylakoids observed in the representative strains of *Phormidiumautumnale*, in the strains of *Microcoleusvaginatus*, the thylakoids were characterised by a fascicular arrangement (Fig. 5).

**Figure 6.**
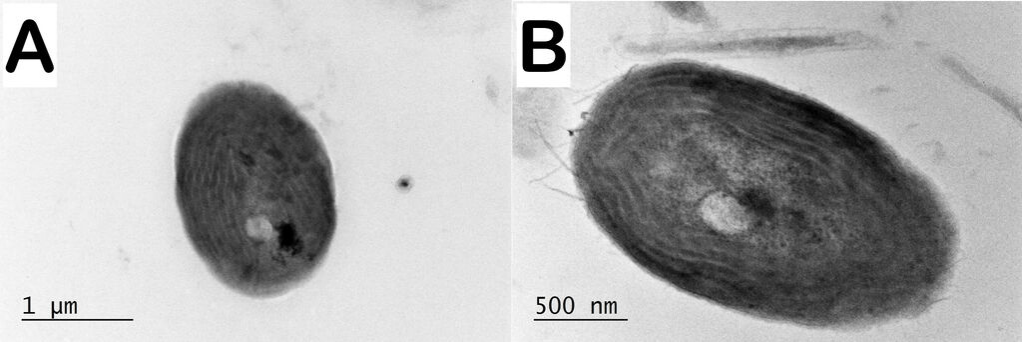
Ultrastructure of *Phormidiumuncinatum* PACC 8693. **A, B** Parietal arrangement of the thylakoids.

Thylakoids in *Phormidiumuncinatum* have also parietal arrangement (Fig. 6).

### Phylogenetic analysis based on 16S rDNA

Phylogenetic reconstructions, based on 16S rDNA (Fig. 7A), showed that investigated *Phormidiumautumnale* strains (PACC 5505, PACC 5511, PACC 5517, PACC 5522, PACC 5527, PACC 5529, marked in bold in the phylogenetic tree) are grouped in a separate clade. This clade was supported by high bootstrap values (0.95/99/68 bootstrap support). The rest of the *Phormidiumautumnale* strains that were used in the phylogenetic analyses formed a sister clade including also other *Phormidium* species. These clades are far from the subtree clearly formed by the representatives of the type species of the genus *Microcoleus*, namely *Microcoleusvaginatus* (Fig. 7B). This is further evidence supporting our hypothesis, based on the morphological and TEM analyses, that *Phormidiumautumnale* has been incorrectly transferred to the genus *Microcoleus* under the name *Microcoleusautumnalis*. In addition, data from the metabolomic analyses also showed significant differences between the investigated *Phormidiumautumnale* and *Microcoleusvaginatus* strains.

The type species of genus *Phormidium* (*Phormidiumlucidum* Kützing ex Gomont, 1892) was grouped together with *Phormidiumchlorinum* (Kutzing ex Gomont 1892) Umezaki and Watanabe 1994 in a distinct clade (Fig. 7A). Taking in account that most oscillatorian genera are polyphyletic, the phylogenetic topology was congruent with the traditional genera defined by morphological features. Aside from *Phormidium* and *Microcoleus*, here we included representatives with the type species of other sister genera belonging to the family Microcoleaceae (*Tychonema*, *Dapis*, *Kamptonema*, *Trichodesmium*, *Arthrospira*) and family Sirenicapillariaceae (*Capilliphycus*, *Neolyngbya*, *Affixifilum*). In addition to genus *Phormidium*, from Oscillatoriaceae were included representatives of genus *Oscillatoria*. Most cyanobacterial strains belonging to one genus were clustered together and formed separate clades. Although the investigated strain *Phormidiumuncinatum* PACC 8693 was clustered within the *Phormidium* clade, its position is not supported by the bootstrap values.

There are currently only two sequences of *Phormidiumpapyraceum* Gomont ex Gomont, 1892 in the GenBank. The BLAST search showed that one of them (OK586776 *Phormidiumpapyraceum* ULC441) has high similarity to strains of Wilmottia murrayi (West & G.S.West) Strunecký, Elster & Komárek 2011 and the other (KF770970 *Phormidiumpapyraceum* PACC 8693) is similar to sequences of *Microcoleusvaginatus* strains. In the reconstructed phylogenetic trees, they are also arranged in such a way.

It was interesting that the other *Microcoleus* species (*M.steenstrupii* J.B. Petersen 1928 and *M.paludosus* Gomont ex Gomont 1892) were clustered together with *Wilmottia* strains, but distinct from the *Microcoleusvaginatus* clade (Fig. 7B). This confirms the note of Komárek & Anagnostidis (Komárek and Anagnostidis 2005) that *Microcoleusvaginatus* should be separated from the genus *Microcoleus* as a special genus, which belongs to the family Oscillatoriaceae.

Distance and similarity between 16S rDNA sequences of the strains used in the phylogenetic analyses are given in Supplementary Materials (Suppl. material 1).

### Metabolomic analysis

To check whether strains belonging to the two genera (*Phormidium* and *Microcoleus*) cultivated under the same conditions differ in their metabolic profile, we performed a metabolomic analysis of non-polar compounds in three *Phormidium* strains (PACC 5522, PACC 5527, PACC 5529) and three strains of *Microcoleus* (CCALA 145, CCALA 152, CCALA 757) by reversed phase chromatography in positive ion mode. The positive ion mode was used as more informative to cover more compounds and provide more comprehensive compound characterisation. We chose to investigate non-polar compounds in order to identify more specific metabolites that could serve as chemo-taxonomic markers for discrimination of strains belonging to these two genera.

**Figure 8.**
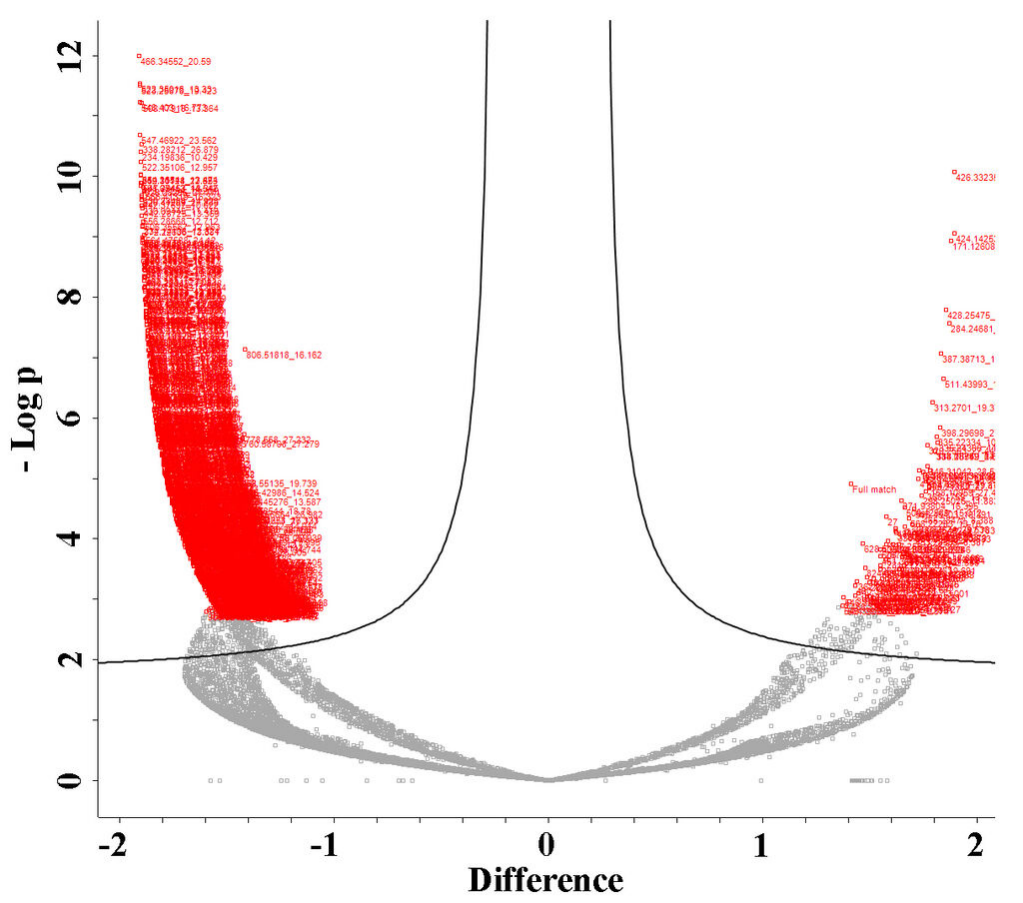
Perseus volcano plot showing compounds with significantly different abundances between the *Phormidium* and *Microcoleus* strains. In the left area, red data are for compounds whose abundances were decreased in *Phormidium* strains and increased in *Microcoleus* strains. In the right side, red data are for compounds with increased abundance levels in *Phormidium* strains and decreased abundance levels in *Microcoleus* strains.

Initial analyses showed the presence of 12,000 potential compounds. After analysis of these compounds with several software packages (including Perseus), 900 compounds were identified that differed significantly between strains of the two genera (Fig. 8).

**Figure 9.**
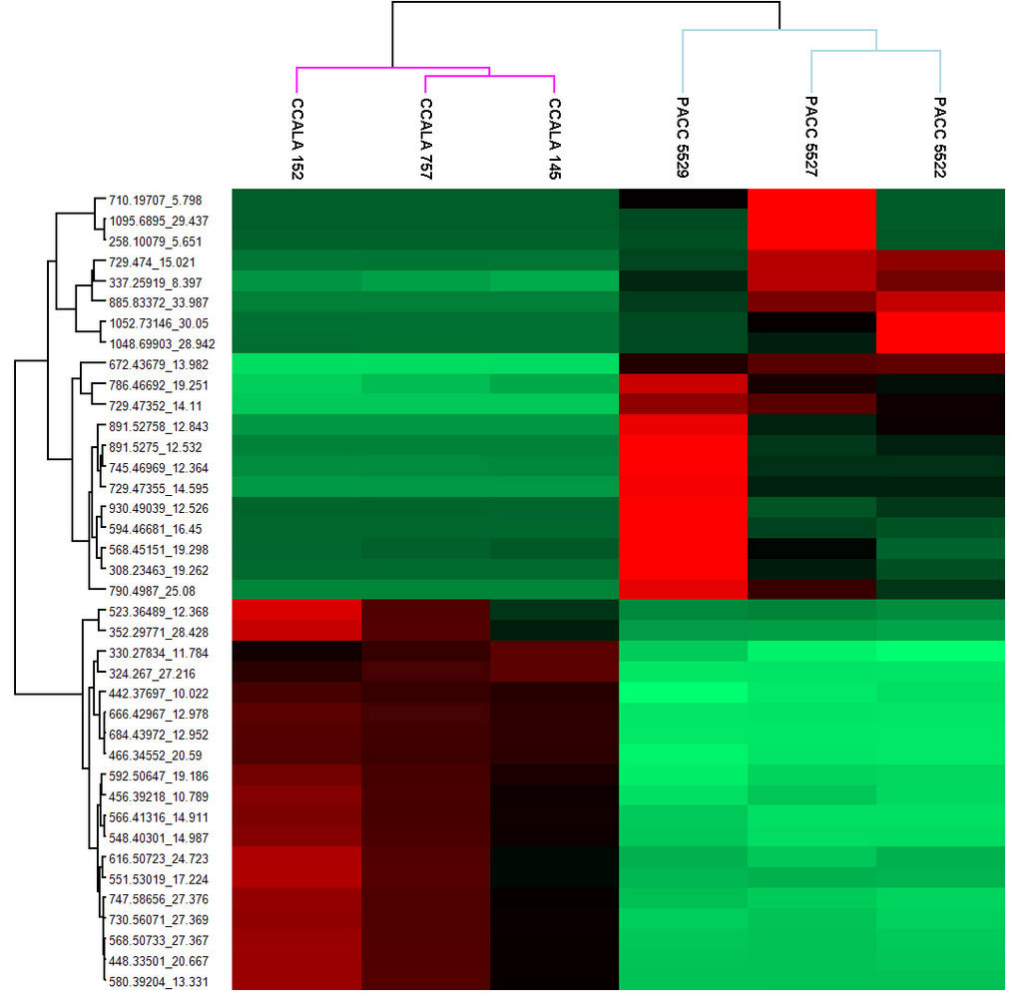
A heatmap of the 39 significant compounds found in the investigated strains designed with the molecular weight and retention time (Y-axis). The six cyanobacterial strains (X-axis) are separated into two groups - *Microcoleus* strains (CCALA 145, CCALA 152, CCALA 757) and *Phormidium* strains (PACC 5522, PACC 5527, PACC 5529). The first 20 compounds (upper part of the Y-axis) are with increased (red) abundance levels in *Phormidium* strains and decreased (green) abundance levels in *Microcoleus* strains. The next 19 compounds (lower part of the Y-axis) are with increased abundance in *Microcoleus* strains and decreased abundance in *Phormidium* strains. The putative identities of these compounds are given in Table 6.

From them, the compounds with the greatest statistical significance were selected for further analysis and identification – a total of 39 in number, 20 with increased concentration and 19 with decreased concentration for the representatives of both genera (Fig. 9). The proposed putative identification is based on three different approaches: Compound Discoverer with FISh coverages, Sirius and MS Finder. Even if not properly annotated, these differences are statistically significant and apparent and the proposed ion features can be used to distinguish between the two cyanobacterial genera (*Phormidium* and *Microcoleus*). Therefore, these 39 compounds presented in Table 6 can be used as potential biochemical markers to distinguish between *Phormidiumautumnale* and *Microcoleusvaginatus*.

## Discussion

A number of morphological and molecular genetic studies have demonstrated the polyphyleticity of the genera *Phormidium* and *Microcoleus* (Komárek et al. 2014, Stoyanov et al. 2014). The genus *Phormidium* presents a significant taxonomic challenge because data obtained with molecular approaches often are inconsistent with the morphological studies. For example, species morphologically assigned to *Phormidiumautumnale* were found to be genetically distinct, grouped into different groups (Marquardt and Palinska 2007).

In addition to the high biodiversity and wide distribution, like most cyanobacteria, the representatives of genus *Phormidium* are also characterised by a high degree of environmentally induced morphological variability (Marquardt and Palinska 2007, Heath et al. 2010). This makes them difficult for identification. Scientific reports clearly show that there is some uncertainty regarding the classification of some members of the genus, such as *P.autumnale* and *P.uncinatum* (McAllister et al. 2016).

Based on molecular genetic analyses, as well as observations on the morphology and ultrastructure of representatives of *Microcoleusvaginatus* and *Phormidiumautumnale*, Strunecký et al. (2013) transfered *P.autumnale* to the genus *Microcoleus* as *Microcoleusautumnalis*. The authors analysed 91 *Microcoleus* strains and only one *Phormidiumautumnale* strain (*M.autumnalis* Luznice). Although this change (based on one strain and literature data) has been accepted, the taxonomic position of *P.autumnale* is still controversial. Proof of this is the data presented in our study, as well as the opinion of other authors who conducted research with *P.autumnale* strains. The polyphasic approach showed that the cyanobacterial blooms observed in New Zealand were due to *P.autumnale* and *P.uncinatum* strains, but molecular genetic analyses, based on 16S rDNA, identified *P.autumnale* as the dominant species (Wood et al. 2012, Harland et al. 2014, McAllister et al. 2016). These findings strongly indicate the need for additional tools to correctly identify the cyanobacterial strains. However, it is obvious that this cannot be accomplished solely through morphology and molecular genetic studies. The inclusion of ultrastructural analysis (e.g. thylakoid arrangement), as well as metabolomic analyzes as additional tools, would, in our opinion, contribute to clarifying this issue.

The polyphasic approach applied in the present study includes a detailed analysis of the morphological features of the two species *Phormidiumautumnale* and *Microcoleusvaginatus*. According to Komárek and Anagnostidis (2005), the genus *Phormidium* contains species with trichomes 3-11 µm width, mucilaginous sheaths with only one trichome, isodiametric cells, shorter than wide, pointed or rounded apical cells with or without calyptra. *Phormidiumautumnale* (Agardh) Trevisan ex Gomont belongs to a group that is characterised by ± isodiametric cells and trichomes that are slightly narrowed at the ends forming calyptra. The morphological characteristics defining genus *Microcoleus* according to Strunecký et al. (2013) are narrowed ends of the trichomes, calyptra, cells shorter than wide, to more or less isodiametric and facultative presence of sheaths. Most species are 4–10 µm in diameter. The presence of multiple trichomes in a common sheath is facultative in many, but not all species.

The main cytomorphological diacritic characters for distinguishing the strains defined in the present study as *P.autumnale* and *M.vaginatus* are: (1) the ends of the trichomes, (2) the shape of the apical cells in the trichome and (3) the presence/absence of a calyptra and its shape (Table 2 and Table 3, Fig. 1 and Fig. 2).

The morphological difference between *Microcoleusvaginatus* and *Phormidiumautumnale* according to Strunecký et al. (2013) is only in the shape of the colonies and the organisation of the filaments. According to data from the same research group, *M.vaginatus* belongs to an easily recognisable clade with a specific ecology (soil biotope) and bundle-like filaments in a common sheath. Our morphological analysis did not confirm these claims. Data showed differences in the morphology of the two strains. In order to avoid the potential influence of other factors on the variability in morphology, the studied cyanobacterial strains were cultured under the same conditions. The ends of the trichomes in the studied *P.autumnale* strains are gradually and slightly narrowed, encompassing the last few cells. In strains of *M.vaginatus*, they are sharply narrowed, S-shaped, with the curve affecting the last few cells and not just the apical one. There is also a difference in the apical cells. In the *P.autumnale* strains, they are elongated, with a rounded conical shape, slightly curved, while in the strains of *M.vaginatus*, the apical cells are capitate. In *P.autumnale*, the calyptra is absent or weakly expressed and, if it is present, it is rounded. In *M.vaginatus* strains, the calyptra is well developed, with a conical, obtuse to hemispherical shape. The filaments are single, which is typical for the genus *Phormidium*. A few trichomes in a common sheath are not observed. There is also a difference in the habitats. *P.autumnale* is a freshwater species distributed mainly in streams, rivers and waterfalls, but also amongst the growths (periphyton) on underwater substrates. The soils are the main habitat for *M.vaginatus*.

Regarding the ultrastructure and thylakoid arrangement, the conclusion of Strunecký et al. (2013) is that the ultrastructure of *P.autumnale* is very similar to that of genus *Microcoleus*. Thylakoids usually form bundle-like aggregations arranged irregularly within the cells. In some strains, there is a radial arrangement of the thylakoids, but in the same strain, the thylakoids may form bundles of thylakoids. We observed that the arrangement of thylakoids in the two species (*P.autumnale* and *M.vaginatus*) shows significant differences. In *P.autumnale*, the thylakoids are with parietal arrangement, sometimes with a central fascicle (Fig. 4) and in *M.vaginatus* strains, the thylakoids are with fascicular arrangement (Fig. 5).

We agree that, due to the high degree of environmentally-induced morphological variability of cyanobacteria, the sequencing is essential for the correct taxonomic assessment of these species. Phylogenetic analyses performed by some research groups suggest that *P.autumnale* is very close to *M.vaginatus* (Boyer et al. 2002, Siegesmund et al. 2008, Hašler et al. 2012, Strunecký et al. 2013). Phylogenetic analyses in the present study confirm the polyphyleticity of genus *Phormidium*, but clearly demonstrate the distance of the clade formed by strains of *Microcoleusvaginatus* from that formed by strains of *Phormidiumautumnale* (Fig. 7A). This is a clear sign of the distance between the two species on a genetic basis, which excludes the identity of *Phormidiumautumnale* with *Microcoleus* strains and its appurtenance to genus *Microcoleus*. Interestingly, the strains we studied showed genetic similarity to representatives of the genus *Kampthonema* separated in 2014 from the genus *Phormidium* (Strunecký et al. 2014). It is clear that, in certain cases, the well-developed and known morphological, ultrastructural and molecular genetic criteria are not sufficiently descriptive and do not provide a definitive answer to the question regarding the taxonomic affiliation and position of a given species. Then it is necessary to look for new characteristics to resolve such an issue.

According to Komárek (2016), differences in biochemistry, for example, in the pigment content and the presence of various compounds (metabolites from the life activity of cyanobacteria), can be specific for different cyanobacterial lineages and could be considered as an additional taxonomic criterion. Some studies have reported the use of fatty acids and lipid profiles of microalgae and cyanobacteria as biomarkers to distinguish closely-related organisms at the species and generic level (Bergé and Barnathan 2005, Schweder et al. 2005, Rossi et al. 2006, Lang et al. 2011). A systematic large-scale analysis of lipid profiles in microalgae was done by Lang et al. (2011), examining all available 2291 microalgal strains of the SAG culture collection. Their conclusion was that, despite the general trends in fatty acid distribution observed throughout the study reflecting the phylogenetic relationships between microalgae species and classes, the fatty acid profile alone cannot be considered as a useful marker for distinguishing between different genera and species. For this purpose, it is necessary to study and compare other metabolites, such as sterols, lipids and hydrocarbons.

The conclusion is that the taxonomic value of various cell inclusions and/or the presence of biochemical compounds is not entirely clear and its evaluation and comparison with other diacritical features in the cyanobacterial taxonomy is needed. To clearly define the taxonomic position of *Phormidiumautumnale*, we performed an additional metabolomic analysis involving three strains of *Phormidiumautumnale* and three strains of *Microcoleusvaginatus*. Based on the analysis, we were able to select 39 compounds that can be used as biochemical markers to distinguish the two species. Our metabolomic analysis clearly showed a different taxonomic affiliation of *Phormidiumautumnale* than that proposed by Strunecký et al. (2013).

The limitations of applying metabolomic analysis within the polyphasic approach as a complementary tool for taxonomic identification are related to the fact that the species being compared must be cultured under the same conditions and cannot be directly applied to natural samples.

## Conclusions

Our results conclusively demonstrate the belonging of the cyanobacterial species *Phormidiumautumnale* to genus *Phormidium* and define its transfer to genus *Microcoleus* as incorrect. Morphological differences were found in the examined *P.autumnale* and *M.vaginatus* strains regarding the ends of the trichome, the shape of the apical cell and the shape of the calyptra, which are sufficiently descriptive. The ultrastructural studies also confirm the differences in the arrangement of thylakoids – parietal in *P.autumnale* and fascicular in *M.vaginatus*. Molecular genetic analyses and phylogenetic reconstructions, based on 16S rDNA, strongly support our opinion that *Phormidiumautumnale* should remain within the genus *Phormidium* and its transfer to the genus *Microcoleus* was incorrect. For the first time, based on a metabolomic analysis, 39 compounds have been selected and proposed as biochemical markers that could serve to distinguish *Phormidiumautumnale* and *Microcoleusvaginatus*.

## Supplementary Material

B3885199-B466-56F8-89FA-3AD39B6AD0AB 10.3897/BDJ.11.e100525.suppl1 Supplementary material 1 Table S1 Data type Distance/Similarity Brief description Polyphasic characterisation of *Microcoleusautumnalis* (Gomont, 1892) Strunecky, Komárek & J.R.Johansen, 2013 (Oscillatoriales, Cyanobacteria) using a metabolomic approach as a complementary tool. File: oo_793915.xlsx https://binary.pensoft.net/file/793915 Ivanka Teneva, Detelina Belkinova, Tsvetelina Paunova-Krasteva, Krum Bardarov, Dzhemal Moten, Rumen Mladenov and Balik Dzhambazov

## Figures and Tables

**Figure 7a.**
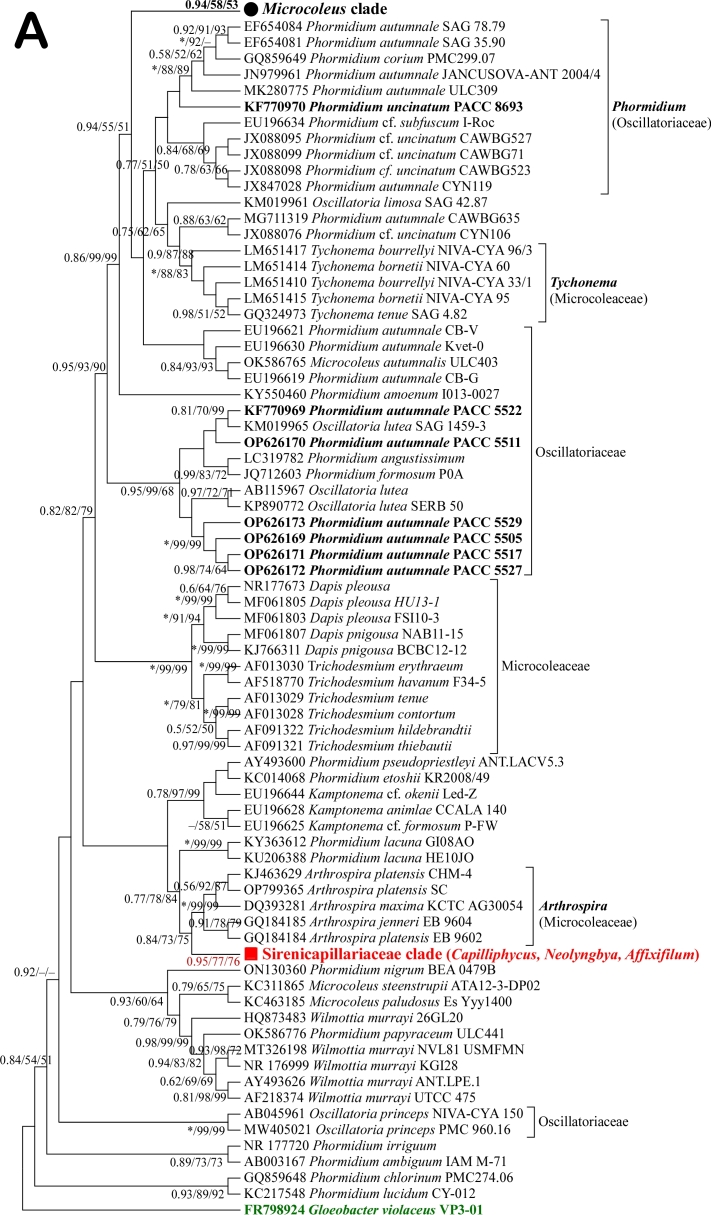


**Figure 7b.**
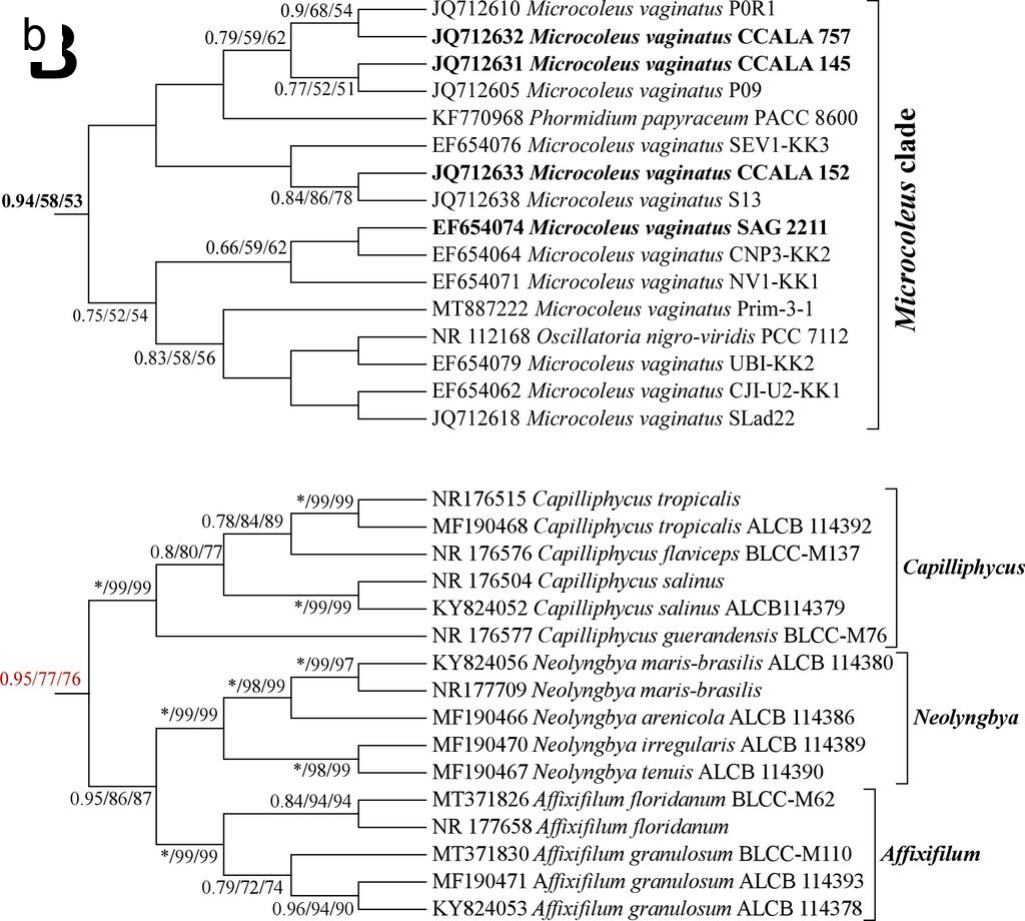


**Table 1.** Origin of the investigated strains.

**Strain**	**Habitat**	**Location**	**Isolated by**
*Phormidiumautumnale* PACC 5505	S-crater Nr. 237	England, Surtsey	Schwabe, 5 Aug 1968
*Phormidiumautumnale* PACC 5511	Lyophilized ampoule	Germany	Steubing, 30 Nov 1967
*Phormidiumautumnale* PACC 5517	Lyophilized ampoule	Germany	Sprecht, 8 Dec 1967
*Phormidiumautumnale* PACC 5522	Moss cultures	Germany	Schwartz-Kraepelin, 21 Nov 1968
*Phormidiumautumnale* PACC 5527	Spillway	Germany, Siegburg	Clasen, 17 Mar 1969
*Phormidiumautumnale* PACC 5529	Meadow	Germany, Solling mountains	Schwabe, 7 May 1968
*Microcoleusvaginatus* CCALA 145	Unknown	Switzerland, Verzascatal	Zehnder, 1964
*Microcoleusvaginatus* CCALA 152	River	Germany, Hamburg	Marvan, 1966
*Microcoleusvaginatus* CCALA 757	Rice field	China, Hubei, Wuhan	Cepak, 1991
*Microcoleusvaginatus* SAG 2211	Soil, desert	USA, New Mexico, Sevielleta LTER	Lewis, Apr 2002
*Phormidiumuncinatum* PACC 8693	Veleka river	Bulgaria, Sinemorets	Mladenov, 5 Oct 1987

**Table 2.** Variability of morphological characters in *Phormidiumautumnale* strains. S – sheath; M – motility; K – keritomy (net-like structure); L/W – mean cell length / mean cell width; (+) – presence; (±) – facultative presence. * According to Mareš et al. (2019).

**Strain**	**S**	**M**	**K**	**L/W**	**Trichome ends**	**Apical cells**	**Calyptra**	**Thylakoid arrangement** *
*Phormidiumautumnale* PACC 5505	±	+	+	0.6	gradually and slightly narrowed	elongated, rounded conical, slightly curved	rounded, weakly expressed	parietal thylakoids with a central fascicle
*Phormidiumautumnale* PACC 5511	±	+	+	0.6	gradually and slightly narrowed	elongated, obtuse-conical, slightly curved	rounded, weakly expressed	parietal thylakoids composed of peripheral fascicles
*Phormidiumautumnale* PACC 5517	±	+	+	0.6	gradually and slightly narrowed	elongated, obtuse-conical, slightly curved	rounded	simple parietal arrangement
*Phormidiumautumnale* PACC 5522	+	+	+	0.8	gradually and slightly narrowed	elongated, rounded conical, slightly curved	truncated	parietal thylakoids with a central fascicle; simple parietal arrangement
*Phormidiumautumnale* PACC 5527	+	+	+	1.0	gradually and slightly narrowed	slightly elongated, curved	truncated or rounded	parietal thylakoids with a central fascicle; simple parietal arrangement
*Phormidiumautumnale* PACC 5529	±	+	+	0.8	gradually and slightly narrowed	elongated, rounded conical, slightly curved	rounded, weakly expressed or absent	parietal thylakoids with a central fascicle

**Table 3.** Variability of morphological characters in *Microcoleusvaginatus* strains. S – sheath; M – motility; K – keritomy (net-like structure); L/W – mean cell length / mean cell width; (+) – presence. * According to Mareš et al. (2019).

**Strain**	**S**	**M**	**K**	**L/W**	**Trichome ends**	**Apical cells**	**Calyptra**	**Thylakoid arrangement** *
*Microcoleusvaginatus* CCALA 145	+	+	+	0.6	abruptly narrowed, curved to S-shaped contorted	capitate	rounded to hemispherical	fascicular arrangement
*Microcoleusvaginatus* CCALA 152	+	+	+	0.5	slightly narrowed, slightly curved	capitate	flat to hemispherical	fascicular arrangement
*Microcoleusvaginatus* CCALA 757	+	+	+	0.6	abruptly narrowed, curved	capitate	hemispherical	fascicular arrangement
*Microcoleusvaginatus* SAG 2211	+	+	+	0.6	abruptly narrowed, curved to S-shaped contorted	capitate	conical, obtuse to hemispherical	fascicular arrangement

**Table 4.** Variability of morphological characters in *Phormidiumautumnale* strains. S – sheath; M – motility; K – keritomy (net-like structure); L/W – mean cell length / mean cell width; (+) – presence; (±) – facultative presence. * According to Mareš et al. (2019).

**Strain**	**S**	**M**	**K**	**L/W**	**Trichome ends**	**Apical cells**	**Calyptra**	**Thylakoid arrangement** *
*Phormidiumuncinatum* PACC 8693	±	+	+	0.3	abruptly narrowed, curved	capitate	rounded conical calyptra	simple parietal arrangement

**Table 5.** Cell sizes of the studied strains. RD – reference data; SD – standard deviation.

**Strain**	**Length of the cells**				**Width of the cells**			
	**Mean (µm)**	**Min (µm)**	**Max (µm)**	**SD**	**Mean (µm)**	**Min (µm)**	**Max (µm)**	**SD**
*Phormidiumautumnale* (RD*)	**2.0–4.0**	–	**5.0**	–	**4.0–7.0**	**3.5**	–	–
*Phormidiumautumnale* PACC 5505	2.5	1.5	4.0	0.7	3.7	3.0	4.0	0.5
*Phormidiumautumnale* PACC 5511	2.4	2.0	3.0	0.5	3.8	3.0	4.0	0.4
*Phormidiumautumnale* PACC 5517	2.3	2.0	3.0	0.5	4.0	3.0	5.0	0.2
*Phormidiumautumnale* PACC 5522	3.1	2.0	5.0	0.6	3.9	3.0	4.5	0.4
*Phormidiumautumnale* PACC 5527	3.2	2.0	6.0	0.9	3.3	2.0	4.0	0.6
*Phormidiumautumnale* PACC 5529	3.0	2.0	5.0	0.6	4.0	3.0	5.0	0.4
*Microcoleusvaginatus* (RD*)	**2.0–5.0**	–	**6.7**	–	**3.0–7.0**	**2.5**	**9.0**	–
*Microcoleusvaginatus* CCALA 145	2.8	2.0	4.0	0.6	4.6	4.0	5.0	0.5
*Microcoleusvaginatus* CCALA 152	2.6	1.0	4.0	0.7	5.6	4.0	7.0	0.7
*Microcoleusvaginatus* CCALA 757	3.0	2.0	4.0	0.7	4.9	4.0	5.0	0.4
*Microcoleusvaginatus* SAG 2211	3.3	2.0	6.0	0.9	5.2	4.0	6.0	0.5
*Phormidiumuncinatum* (RD*)	**2.0–6.0**	**2.0**	**6.0**	–	**5.5–9.0**	**4.0**	**9.5**	–
*Phormidiumuncinatum* PACC 8693	2.6	1.0	4.0	0.6	7.5	6.0	9.0	0.7

* Komárek & Anagnostidis (2005).

**Table 6.** Biochemical markers for distinguishing *Phormidiumautumnale* and *Microcoleusvaginatus*. RT, retention time; ­­­­(+) increased abundance; (–) decreased abundance.

**No**	**RT** **(min)**	**Compound**	**Formula**	**Molecular weight**	***P.autumnale***	***M.vaginatus***
1	5.65	6-Ethyl-2-methyl-4,6-dihydro-2H-[1,4]oxazino[3,2-c]quinoline-3,5-dione	C_14_H_14_N_2_O_3_	258.101	+	–
2	5.80	Unknown	C_32_H_40_N_8_OP_2_S_3_	710.197	­+	–
3	8.40	Unknown	C_17_H_37_O_6_	337.259	­+	–
4	10.02	Ethyl N-{2-[(tert-butoxycarbonyl)amino]hexadecyl}glycinate	C_25_H_50_N_2_O_4_	442.377	–	­+
5	10.79	1,16-Hexadecanediyl bis(butylcarbamate)	C_26_H_52_N_2_O_4_	456.392	–	­+
6	11.78	2-Palmitoylglycerol	C_19_H_38_O_4_	330.278	–	­+
7	12.36	6-Hydroxy-9-[(6Z,9Z,12Z,15Z)-6,9,12,15-octadecatetraenoyloxy]-6-oxido-5,7-dioxa-2-aza-6lambda~5~-phosphadecan-10-yl (6Z,9Z,12Z,15Z)-6,9,12,15-octadecatetraenoate	C_42_H_68_NO_8_P	745.470	­+	–
8	12.37	(3R)-3-{[(3alpha,5beta)-3-Hydroxy-24-oxocholan-24-yl]amino}-3-phenylpropanoic acid	C_33_H_49_NO_4_	523.365	–	­+
9	12.53	5-Oxo-L-prolyl-L-threonyl-L-seryl-L-phenylalanyl-L-threonyl-L-prolyl-N~5~-(diaminomethylene)-L-ornithyl-L-leucinamide	C_42_H_66_N_12_O_12_	930.490	­+	–
10	12.53	Unknown	C_49_H_74_N_5_O_8_P	891.528	­+	–
11	12.84	Unknown	C_49_H_74_N_5_O_8_P	891.528	­+	–
12	12.95	(3beta,22beta)-22-[(3-Methyl-2-butenoyl)oxy]-3-{[(2E)-3-phenyl-2-propenoyl]oxy}olean-12-en-28-oic acid	C_44_H_60_O_6_	684.440	–	­+
13	12.98	4-Methyl-6-oxostigmast-7-ene-3,22-diyl dibenzoate	C_44_H_58_O_5_	666.430	–	­+
14	13.33	Phoenicoxanthin	C_40_H_52_O_3_	580.392	–	­+
15	13.98	1-Ethyl-4-(4-oxido-2,6-diphenyl-4H-1,4-oxaphosphinin-4-yl)piperazine	C_36_H_72_N_3_O_4_PS	672.437	­+	–
16	14.11	Methyl N-[(3beta)-3,23-dihydroxy-28-oxolup-20(29)-en-28-yl]glycyl-L-tryptophanate	C_44_H_63_N_3_O_6_	729.474	­+	–
17	14.60	Methyl N-[(3beta)-3,23-dihydroxy-28-oxolup-20(29)-en-28-yl]glycyl-L-tryptophanate	C_44_H_63_N_3_O_6_	729.474	­+	–
18	14.91	3-Hydroxyechinenone	C_40_H_54_O_2_	566.413	–	­+
19	14.99	(3'Z)-3',4'-Didehydro-beta,psi-caroten-4-one	C_40_H_52_O	548.403	–	­+
20	15.02	Methyl N-[(3beta)-3,23-dihydroxy-28-oxolup-20(29)-en-28-yl]glycyl-L-tryptophanate	C_44_H_63_N_3_O_6_	729.474	­+	–
21	16.45	Unknown	C_33_H_63_N_4_O_3_P	594.467	­+	–
22	17.22	N-heptadecanoylsphingosine	C_35_H_69_NO_3_	551.530	–	­+
23	19.19	1-Palmitoyl-2-linoleoyl-sn-glycerol	C_37_H_68_O_5_	592.506	–	­+
24	19.25	L-Phenylalanyl-L-leucyl-L-arginyl-L-isoleucyl-L-arginyl-L-prolyl-L-lysine	C_34_H_73_N_6_O_8_P_3_	786.467	­+	–
25	19.26	Eicosapentaenoic acid methyl 9-oxooctadeca-10,12-dienoate	C_19_H_32_O_3_	308.235	­+	–
26	19.30	[5-(5a,5b,8,8,11a,13b-Hexamethyl-1,2,3,3a,4,5,7a,9,10,11,11b,12,13,13a-tetradecahydrocyclopenta[a]chrysen-3-yl)-2-acetyloxyhexyl] acetate	C_38_H_56_N_4_	568.452	­+	–
27	20.59	Phylloquinone oxide	C_31_H_46_O_3_	466.346	–	­+
28	20.67	2-Methyl-2-[(3E,7E,11E)-4,8,12,16-tetramethyl-3,7,11,15-heptadecatetraen-1-yl]-2H-chromen-6-ol	C_31_H_44_O_2_	448.335	–	­+
29	24.72	1-Palmitoyl-2-arachidonoyl-sn-glycerol	C_39_H_68_O_5_	616.507	–	­+
30	25.08	(1R,2R,3S,4R,6S)-4,6-diamino-2-{[(2R,15R)-16-({(1R,2R,3S,5R,6S)-3,5-diamino-2-[(2,6-diamino-2,6-dideoxy-alpha-D-glucopyranosyl)oxy]-6-hydroxycyclohexyl}oxy)-2,15-dihydroxy-4,13-dimethyl-7,10-dioxa-4,13-diazahexadec-1-yl]oxy}-3-hydroxycyclohexyl 2,6-diamino-2,6-dideoxy-alpha-D-glucopyranoside	C_43_H_87_N_2_O_8_P	790.499	­+	–
31	27.22	Ethyl (9E)-8-oxo-9-octadecenoate	C_20_H_36_O_3_	324.267	–	­+
32	27.37	[(2S)-2-hexadecanoyloxy-3-hydroxypropyl] hexadecanoate	C_35_H_68_O_5_	568.507	–	­+
33	27.37	1,2-Dipalmitoyl-3-beta-D-galactosyl-sn-glycerol	C_41_H_78_O_10_	730.561	–	­+
34	27.38	(2R)-N-[(2S,3S,4R)-1-(beta-L-Allopyranosyloxy)-3,4-dihydroxy-2-undecanyl]-2-hydroxytetracosanamide	C_41_H_81_NO_10_	747.587	–	­+
35	28.43	3-Octadecyloxolane-2,5-dione	C_22_H_40_O_3_	352.298	–	­+
36	28.94	Unknown	C_63_H_98_N_6_OP_2_S	1048.699	­+	–
37	29.44	(2R)-N-[(2S,3R,5E)-1,3-Dihydroxy-5-heptadecen-2-yl]-2-hydroxyicosanamide	C_53_H_106_N_9_O_3_P_3_S_2_	1095.690	­+	–
38	30.05	Unknown	C_69_H_101_N_2_O_2_PS	1052.731	­+	–
39	33.99	O-[{(2R)-3-[(13Z,16Z)-13,16-Docosadienoyloxy]-2-[(4Z,7Z,10Z,13Z,16Z,19Z)-4,7,10,13,16,19-docosahexaenoyloxy]propoxy}(hydroxy)phosphoryl]-L-serine	C_47_H_90_N_2_O_13_	885.834	­+	–
